# Use of thrombocyte count dynamics after aneurysmal subarachnoid hemorrhage to predict cerebral vasospasm and delayed cerebral ischemia: a retrospective monocentric cohort study

**DOI:** 10.1038/s41598-025-93767-y

**Published:** 2025-03-21

**Authors:** Jan Oros, Stefanos Voglis, Ferdinand Oliver Bohmann, Lina Elisabeth Qasem, Christophe Théo Arendt, Fee Keil, Wolfgang Miesbach, Marcus Czabanka, Sarah Christina Reitz

**Affiliations:** 1 Center for Neurology and Neurosurgery, Department of Neurosurgery, Goethe University Frankfurt, University Hospital, Frankfurt am Main, Germany; 2 Department of Neurosurgery, University of Zürich, University Hospital, Zürich, Switzerland; 3 Center for Neurology and Neurosurgery, Department of Neurology, Goethe University Frankfurt, University Hospital, Frankfurt am Main, Germany; 4 Institute of Neuroradiology, Goethe University Frankfurt, University Hospital, Frankfurt am Main, Germany; 5 Department of Hematology and Oncology, Goethe University Frankfurt, University Hospital, Frankfurt am Main, Germany

**Keywords:** Aneurysmal subarachnoid hemorrhage, Cerebral vasospasm, Delayed cerebral ischemia, Thrombocyte dynamics, Neurology, Neurological disorders, Cerebrovascular disorders, Stroke, Neurological disorders, Cerebrovascular disorders, Stroke

## Abstract

Cerebral vasospasm (CVS) and delayed cerebral ischemia (DCI) are critical complications following aneurysmal subarachnoid hemorrhage (aSAH), contributing to substantial morbidity and mortality. This retrospective cohort study investigated thrombocyte count (TC) dynamics as a potential marker for predicting CVS and DCI in 233 adult patients with aSAH. Parameters including TC, C-reactive protein, hematocrit, CVS, and DCI were analyzed using logistic regression, Spearman correlation, and time-to-event analysis. CVS and DCI occurred in 71.1% and 41.2% of patients, respectively. A relative thrombocyte count decrease greater than 12.6% within the early post-aSAH period was significantly associated with increased risks of CVS (*p* < 0.001; 95% CI 4.74–25.3) and DCI (*p* = 0.003; 95% CI 1.39–5.43). Temporal analysis revealed that greater TC decrease correlated with earlier CVS onset (*p* = 0.00016; *R*=-0.28), with a median of three days from the minimum TC to CVS onset. This association suggests a potential diagnostic window for early detection and intervention if validated in prospective studies.

**Supplementary Information:**

The online version contains supplementary material available at 10.1038/s41598-025-93767-y.

## Introduction

CVS and DCI remain the leading causes of morbidity and mortality aSAH^1^. The current literature reports incidences of CVS and DCI as high as 70% and 30%, respectively^2,3^. DCI is linked with poor functional and cognitive outcomes, severe disability, and a higher mortality rate^4^. The accurate prediction of CVS and/or DCI remains a significant challenge in clinical practice. Transcranial Doppler sonography (TCD), a widely used noninvasive screening method for CVS, is not directly applicable to the screening of DCI and is associated with high interobserver variability^5,6^. The reliability of partial tissue oxygen pressure (ptO_2_) measurements is highly dependent on the region of implantation^7,8^, and microdialysis largely remains an experimental method^9^. This underscores the necessity for more dependable predictors that can anticipate the onset of CVS/DCI at its earliest stages, thereby enabling prompt therapeutic intervention.

The precise pathophysiology of CVS and DCI has been a subject of both basic and clinical research for decades. The processes leading to the development of CVS appear to be an interplay of mechanisms, including endothelial damage^10–12^, the release of vasoconstrictive agents and lack of vasodilatory agents^10,12–15^, the disruption of microcirculation^16,17^, and spreading depolarizations^18^. Some of these mechanisms can be directly or indirectly regulated, or even dysregulated, by thrombocytes, which are the main mediators of primary hemostasis. The upregulation, downregulation, and release of vasoactive agents by thrombocytes have been the subject of several studies on the development of CVS and DCI^19,20^. Additionally, rapid thrombocyte aggregation leading to thrombocyte depletion hours after aSAH has been demonstrated in several animal models^21,22^.

The objective of this study was to investigate the association between a relative decrease in plasma thrombocyte count (TC) shortly after aSAH and the development of CVS and DCI.

## Methods

### Study design and data collection

This study was conducted as a retrospective cohort study. All patients with aSAH admitted to our Center for Neurology and Neurosurgery (Goethe University Frankfurt, University Hospital, Frankfurt am Main, Germany) between January 2015 and May 2023 were screened for eligibility. This study was approved by the Goethe University Hospital Ethics Committee (approval no. 2023 − 1408). Data were retrieved from a mandatory aSAH inpatient quality assurance registry. Our study was designed in accordance with relevant guidelines and regulations and followed the principles of the Declaration of Helsinki. The following parameters were assessed: age, sex, date of admission, date of discharge, time of approximate symptom onset, type of therapy, and the presence of CVS. CVS was defined as radiographically diagnosed CVS via computer tomographic angiography (CTA), magnetic resonance angiography (MRA), or digital subtraction angiography (DSA) that prompted the induced hypertonic therapy, the presence of DCI, defined as radiographically proven hemodynamic infarctions as judged by a board-certified neuroradiologist; was also considered. To classify the severity of aSAH according to the modified Fisher scale (mFS)^23^, archived cranial CT scans (cCT) from the day of admission were reviewed. The approximate time point of symptom onset was marked as day 0. Setting this timepoint as the baseline, laboratory results of thrombocyte count (TC), C-reactive protein (CRP), and hematocrit (Hct) between day 1 and day 7 were analyzed. The lowest recorded TC between day 1 and day 7 was noted, along with the day on which it occurred (hereafter referred to as “day TCmin”). To identify the relative TC difference, the TC value on day 1 was subtracted from the lowest TC value recorded. All included patients were screened for intake of antiplatelet medication and anticoagulation. The intra-interventional administration of these agents was also noted. Patients’ medical histories were reviewed for hereditary platelet conditions as well as autoimmune and atopic diseases.

All recorded data were collected in accordance with the General Data Protection Regulation. Based on § 27 BDSG (Art. 9 GDPR, § 2 (j) https://gdpr-info.eu/art-9-gdpr/) considering the exceptions to GDPR regulations for scientific purposes, the obtainment of the informed consent was waived by the Goethe University Hospital Ethics Committee.

All patients received a standardized intensive care bundle in accordance with our internal standard operating procedure for aSAH. This included invasive blood pressure measurement, daily oral administration of nimodipine from day 0 to day 21 after aSAH, CVS screening, which entailed daily TCD, post-clipping DSA, weekly CT-A and/or CT-A in the event of clinical/ultrasonographic suspicion of CVS, and the initiation of induced hypertension therapy following radiographic evidence of CVS. The decision which treatment modality was selected to treat our patients was made in an interdisciplinary fashion by senior physicians in the Department of Neurosurgery and Department of Neuroradiology. All patients undergoing endovascular treatment received intravenous weight-adjusted unfractionated heparin (UFH) infusion. Patients without acetylsalicylic acid in their home medication also received intravenous ASA infusion.

The two pre-specified primary outcomes were the development of CVS and/or DCI.

### Inclusion and exclusion criteria

**Fig. 1:**
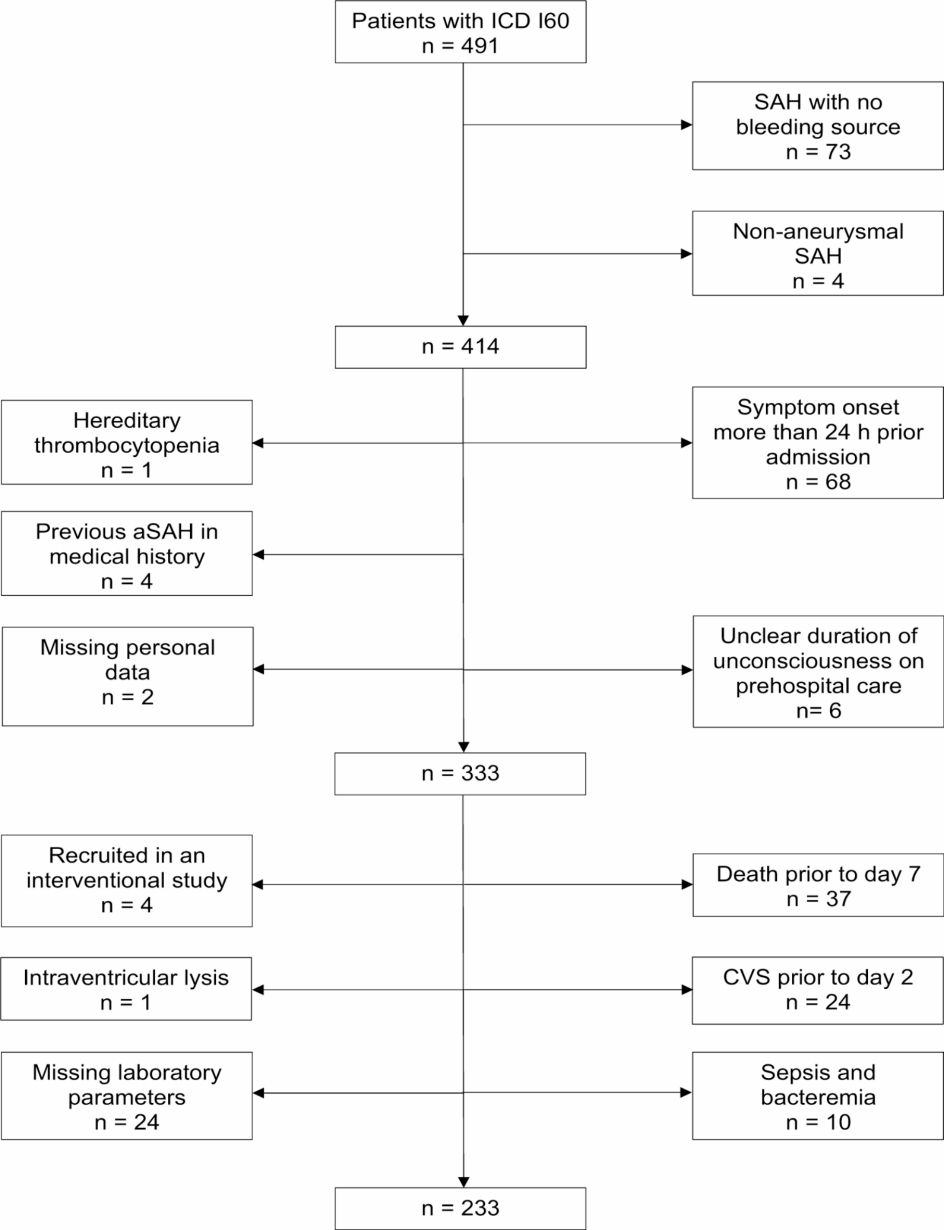
Flow chart of patient selection process. *ICD* international classification of diseases, *SAH* subarachnoid hemorrhage, *CVS* cerebral vasospasm.

Following the initial screening of our electronic medical records, a total of 491 adult patients with SAH (ICD-10 code I60) were identified. Patients with non-aneurysmal SAH and patients in whom no bleeding source was found were excluded. Only adult patients admitted no later than 24 h after symptom onset were included. This criterion was set to allow monitoring of TC as early as possible. Patients with conditions significantly influencing the plasma TC, such as hereditary TC conditions, intraventricular lysis, or sepsis, were excluded. Additionally, patients were excluded if their personal data were missing, laboratory results were missing on days of interest, or the duration of loss of consciousness before admission was unclear. In 24 patients (5.4% of all screened aSAH patients), CVS were detected prior to day 2 after aSAH. This would suggest that the initial onset of aSAH might be more than 24 h prior to admission, which would render our capacity to detect an early decrease in thrombocyte count as our primary independent variable of interest impossible. Moreover, some studies consider ultra-early CVS (under 48 h) as a separate entity due to their higher incidence of symptomatic CVS and DCI^24,25^. We identified this as a potential bias, which led to the exclusion of these patients. Furthermore, participation in interventional studies rendered these patients ineligible for this study due to the potential for bias. After applying the aforementioned exclusion criteria, 233 patients were included for analysis. A study flowchart is shown in Fig. 1.

### Statistical analysis

**Fig. 2:**
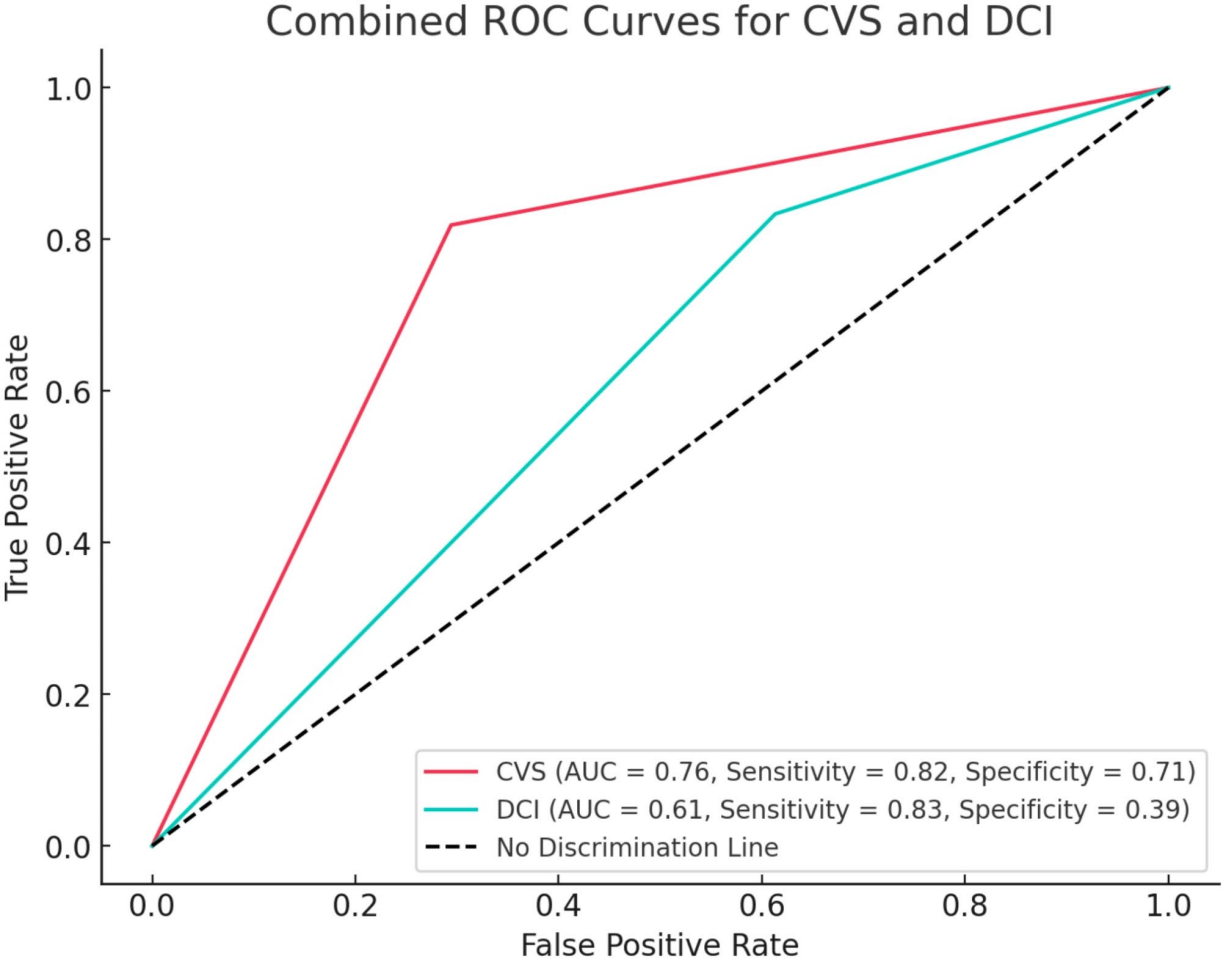
The figure displays the ROC curves for the TC decrease > 12.6% in predicting CVS and DCI. The red curve represents CVS with an AUC of 0.76, sensitivity of 81.87%, and specificity of 70.59%. The teal curve represents DCI with an AUC of 0.61, sensitivity of 83.33%, and specificity of 38.69%. The dotted diagonal line represents no discrimination. *ROC* receiver operating curves, *AUC* area under the curve, *CVS* cerebral vasospasm, *DCI* delayed cerebral ischemia.

Statistical analysis was conducted using RStudio version 4.3.0 (RStudio: Integrated Development for R. RStudio, PBC, Boston, MA). First, descriptive statistics were employed to identify the basic characteristics that distinguished four groups of patients based on the presence or absence of CVS and DCI. For continuous data, the median with interquartile range (IQR) was calculated (median [25th quartile; 75th quartile]). The Wilcoxon matched-pairs test was employed to compare the median laboratory values between day 1 and day 7 in patients with and without CVS and DCI as dependent variables. Univariate logistic regression was conducted to identify independent predictors of CVS and DCI development, followed by an adjusted multivariate logistic regression with the inclusion of variables regardless of statistical significance in the univariate analysis. Maximally selected log-rank statistics determined the optimal cut-off value of the relative TC decrease for the stratification of the time-to-event analysis (time-to-CVS). Implementing the cut-off of the relative TC decrease, an additional binary multivariate logistic regression on the development of CVS and DCI was performed. Only variables that demonstrated significant predictive value in a univariate setting were included in this analysis. ROC analysis was performed to assess the predictive value of our independent variable of interest. Spearman correlation was used to investigate the relationship between the extent of the observed TC decrease and the timing of CVS. Finally, additional univariate and multivariate logistic regressions on the development of DCI in a subgroup of patients with CVS were performed.Fig. 2

## Results

### Patient cohort characteristics

Of the 233 patients with aSAH enrolled, 182 (71.1%) developed CVS and 96 (41.2%) developed DCI. This accounted for 95 (52%) patients with CVS (*p* < 0.001). The median age was 56 [49; 65] years. Among the CVS patients, 94 (52%) underwent surgical clipping. In patients without CVS, the clipping procedure was performed in 18 cases (35%) (*p* = 0.039). In patients with DCI, 54 (56%) underwent surgical clipping, whereas in patients without DCI, 58 (42%) (*p* = 0.036) were treated surgically. All basic characteristics are summarized in Table 1.

**Table 1:** Basic characteristics grouped by the presence of CVS and DCI.

Characteristics	*n* = 233^*1*^	Cerebral vasospasm		*p*-value^2^	Delayed cerebral ischemia		
		no, *n* = 51 (21.9%)^*1*^	yes, *n* = 182 (71.1%)^*1*^		no, *n* = 137 (58.8%)^1^	yes, *n* = 96 (41.2%)^1^	*p*-value^2^
Sex				0.9			0.6
female	153 (66%)	34 (67%)	119 (65%)		88 (64%)	65 (68%)	
male	80 (34%)	17 (33%)	63 (35%)		49 (36%)	31 (32%)	
Age	57.1 (13.3)	59.3 (16.8)	56.5 (12.1)	0.3	57.56 (13.9)	56.4 (12.4)	0.6
Therapy				0.039*			0.036*
endovascular	121 (52%)	33 (65%)	88 (48%)		79 (58%)	42 (44%)	
surgical	112 (48%)	18 (35%)	94 (52%)		58 (42%)	54 (56%)	
DCI				< 0.001**			N/A
yes	96 (41%)	1 (2.0%)	95 (52%)		N/A	N/A	
no	137 (59%)	50 (98%)	87 (48%)		N/A	N/A	
mFS				< 0.001**			0.11
1	20 (8.6%)	11 (22%)	9 (4.9%)		15 (11%)	5 (5.2%)	
2	13 (5.6%)	2 (3.9%)	11 (6%)		6 (4.4%)	7 (7.3%)	
3	121 (52%)	31 (61%)	90 (49%)		76 (55%)	45 (47%)	
4	79 (34%)	7 (14%)	72 (40%)		40 (29%)	39 (41%)	

^1^Statistics presented: n (%) and median with standard deviation (SD).

^2^Pearson’s Chi-squared test (for all *n* ≥ 5) and Fisher’s exact test (for all *n* < 5) for categorical and Wilcoxon rank sum test for continuous variables.

* *p* < 0.05; ** *p* < 0.001.

*CVS* cerebral vasospasm, *DCI* delayed cerebral ischemia, *mFS* modified fisher scale.

### The dynamics of the laboratory parameters in patients with and without CVS and DCI

**Fig. 3:**
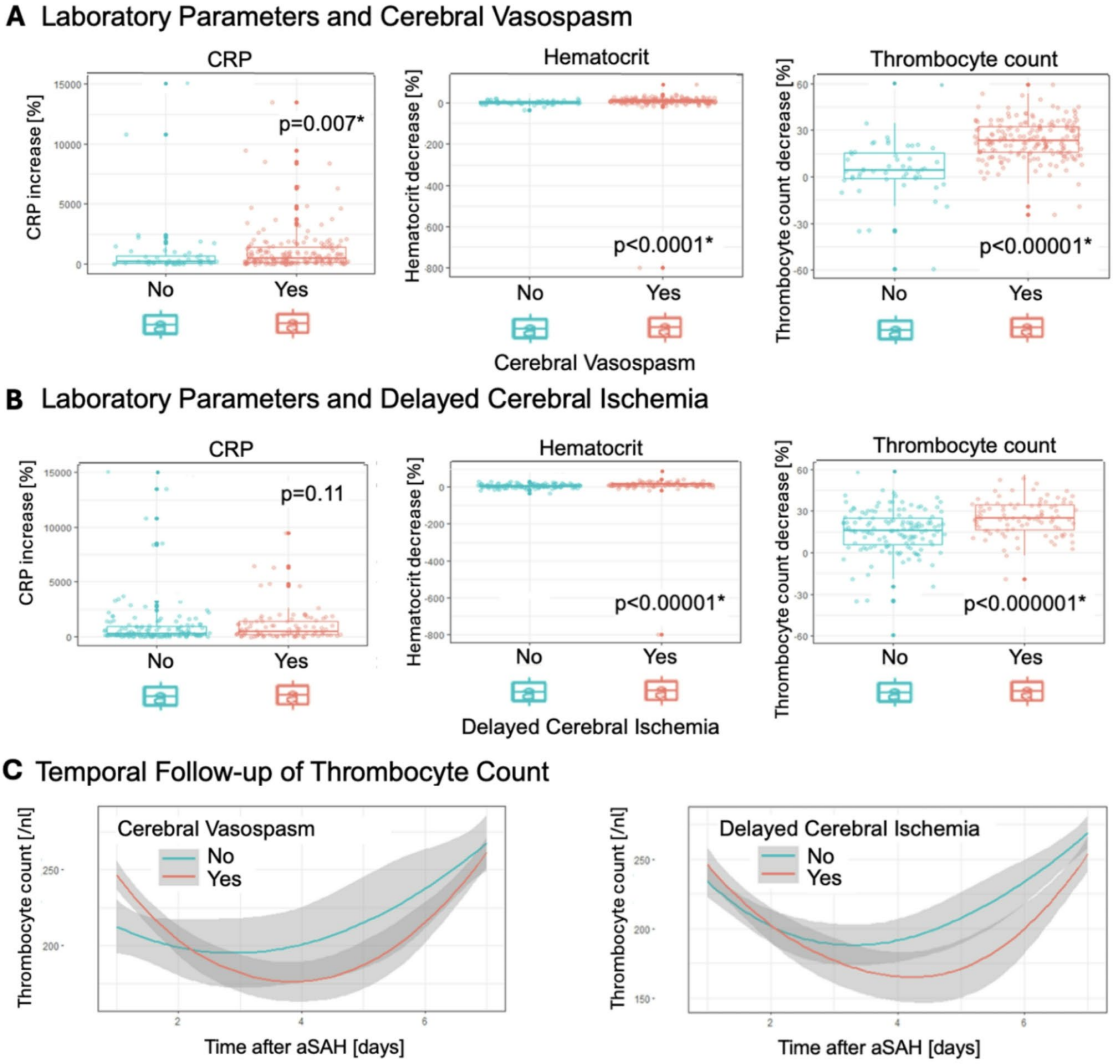
(**A**): Box plots depicting the results of Wilcoxon matched pair test showing significant differences in percentual changes of all three laboratory parameters grouped by CVS presence (no CVS = blue, CVS = red). Median, IQR with 25th and 75^Th^ quartile and maximums without outliers are presented. This suggests heterogeneity between patients with and without CVS in these parameters. This was most distinct for the changes in TC. (**B**): Box plots depicting the results of Wilcoxon matched pair test showing differences in percentual changes of laboratory parameters grouped by DCI presence (no DCI = blue, DCI = red). Median, IQR with 25th and 75^Th^ quartile and maximums without outliers are presented. Only Hct and TC values showed significant differences, with TC showing the most significant distinction.(**C**): Temporal follow-up [days after aSAH] of median TC values [/nl] with 95% CI grouped by CVS and DCI presence (no CVS/DCI = blue line, CVS/DCI = red line; line represents median) showing steeper decrease of TC during the first two days after aSAH in patients with CVS and DCI. In patients with CVS and DCI overall lower TC values were reached. *CRP* C-reactive protein, *Hct* hematocrit, *TC* thrombocyte count, *CVS* cerebral vasospasm, *CI* confidence interval, *IQR* interquartile range, *DCI* delayed cerebral ischemia.

In patients with CVS, the median relative difference of TC between day 1 and the day on which the minimum TC was observed was − 23.3% [-32.3; -15.7], compared to -4.3% [-15.3; 1.3] in patients without CVS (*p* < 0.0001). Patients with CVS exhibited a median increase in CRP of 495.5% [152.5; 1392], while patients without CVS demonstrated a median increase of 211% [59.1; 669.4] (*p* = 0.007). Significant differences were also observed in median Hct decrease between patients with and without CVS. The former exhibited a median Hct decrease of -8.1% [-16.2; -3.4], while the latter exhibited a median Hct decrease of -2.3% [-7.6; 3.4] (*p* < 0.0001) (Fig.3). The temporal follow-up of TC in patients with and without CVS is shown in Fig. 3C.

Similar to CVS, significant differences in median TC decrease were also observed between patients with and without DCI: -24.8% [-34.3; -16.6] vs. 16.1% [-24.7; -5.9] (*p* < 0.0001), respectively. The median Hct decrease also demonstrated significant differences between patients with and without DCI: -10.6% [-18.6; -5.3] vs. 5.4% [-10.3; 0.6] (*p* < 0.0001). There were no significant differences in median CRP increase between patients with and without DCI (Fig. 3B). Figure 3 C illustrates the temporal follow-up of the TC in patients with and without DCI.

### Thrombocyte count decrease early after aSAH associated with increased risk of CVS and DCI

A significant univariate association was found between the relative TC decrease and the development of CVS (OR 1.11, CI [1.07–1.15], *p* < 0.001). Patients with higher grades on mFS exhibited a significantly increased risk of CVS (mFS 4 vs. mFS 1: OR 12.6, CI [4.01–43], *p* < 0.001). Additionally, endovascularly treated patients demonstrated a lower risk of CVS (OR 0.52, CI [0.27–0.98], *p* < 0.001). The development of CVS was not linked with age, sex, Hct, or CRP levels (Table 2).

In our multivariate model, the relative TC decrease shortly after aSAH was linked with higher risk of CVS (OR 1.1, CI [1.06–1.14], *p* < 0.001). Higher mFS grades were found to be significantly predictive of CVS (mFS 4 vs. mFS 1: OR 23.1, CI [5.21–117], *p* < 0.001). Furthermore, endovascular treatment was found to remain associated with a smaller relative risk of CVS (OR 0.4, CI [0.16–0.96], *p* = 0.039). The remaining variables demonstrated no significant association with CVS (Table 2).

A higher risk of DCI was observed in patients with a relative TC decrease in the univariate model (OR 1.05, CI [1.03–1.08], *p* < 0.001). The association of endovascular therapy with a lower relative risk of DCI was observed only in the univariate analysis (OR 0.57, CI [0.34–0.96], *p* = 0.036). In a multivariate analysis, the only single predictor significantly associated with the development of DCI was the relative TC decrease (OR 1.05, CI [1.03–1.08], *p* < 0.001) (Table 2).

**Table 2:** Prediction of CVS and DCI in univariate and multivariate logistic regression.

	Cerebral Vasospasm						Delayed cerebral Ischemia					
	Univariate logistic regression			Multivariate logistic regression			Univariate logistic regression			Multivariate logistic regression		
*n* = 233	OR	95% CI	p-value	OR	95% CI	p-value	OR	95% CI	p-value	OR	95% CI	p-value
Age	0.98	0.96, 1.01	0.18	0.99	0.96, 1.02	0.55	0.99	0.97, 1.01	0.49	0.99	0.97, 1.01	0.47
Sex			0.86			0.26			0.58			0.47
female	—	—		—	—		—	—		—	—	
male	1.06	0.55, 2.08		0.59	0.23, 1.47		0.86	0.49, 1.48		0.79	0.41, 1.5	
Therapy			0.037*			0.039*			0.036*			0.23
endovascular	0.51	0.26, 0.96		0.4	0.16, 0.95		0.57	0.34, 0.96		0.69	0.37, 1.27	
surgical	—	—		—	—		—	—		—	—	
mFS			< 0.001**			< 0.001**			0.11			0.58
1	—	—		—	—		—	—		—	—	
2	6.72	1.35, 51.3		5.08	0.61, 65.2		3.50	0.81, 16.6		2.04	0.40, 11.1	
3	3.55	1.35, 9.6		6.35	1.85, 22.8		1.78	0.64, 5.76		1.62	0.54, 5.56	
4	12.6	4.01, 43		23.8	5.34, 121		2.93	1.02, 9.7		2.18	0.68, 7.9	
TC decrease [%]	1.11	1.07, 1.15	< 0.001**	1.10	1.06, 1.14	< 0.001**	1.05	1.03, 1.08	< 0.001**	1.05	1.03, 1.08	< 0.001**
Hct decrease [%]	1	0.99, 1.01	0.75	1		0.50	1	0.99, 1.01	0.89	1	0.99, 1.01	0.74
CRP increase [%]	1	1, 1	0.64	1	1, 1	0.45	1	1, 1	> 0.99	1	1, 1	0.68

* *p* < 0.05; ** *p* < 0.001.

*CVS* cerebral vasospasm, *DCI* delayed cerebral ischemia, *mFS* modified fisher scale, *TC* thrombocyte count, *Hct* hematocrit, *CRP* C-reactive-protein.

### Thrombocyte count decrease is associated with shorter CVS-free intervals after aSAH, and its extent correlates with earlier onset of CVS

**Fig. 4:**
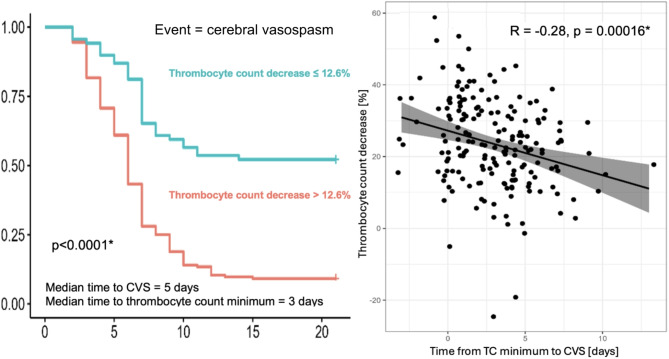
Left: Kaplan-Meier curve showing significantly lower probability (*p* < 0.0001) of CVS-free interval in aSAH patients with TC decrease > 12.6% (red line) as compared to patients with TC decrease ≤ 12.6% (blue line). * marks significant results.

After determining the optimal cut-off of 12.6% for relative TC decrease, the time-to-event analysis was performed. In patients with a TC decrease above the 12.6% cut-off, 18.9% showed no evidence of CVS on day 10 compared to 59.4% CVS-free patients in the group without such a TC decrease. Only 9.8% of patients with a TC decrease above the cut-off remained without CVS on day 15 after aSAH compared to 52.2% of patients below the cut-off, showing that the group of patients with TC decrease above 12.6% showed more than 40% a higher event rate compared to patients without such TC decrease (*p* < 0.0001) (Fig.4 left).

By correlating the extent of TC decrease with the timing of CVS onset, we observed earlier CVS in patients with more extensive TC decrease (*R* = -0.28, *p* = 0.00016) (Fig.4, right).

### The thrombocyte count decrease above 12.6% confers a 10-fold increased risk of CVS and a 3-fold increased risk of DCI in both surgically and endovascularly treated patient

After dichotomizing our cohort into patients with and without TC decrease based on the 12.6% cut-off, we performed additional multivariate logistic regression. Patients with a TC decrease greater than 12.6% had a high relative risk of developing CVS (OR 10.6, CI [4.74–25.3], *p* < 0.001) and DCI (OR 2.7; CI [1.39–5.43]) (Table3. These observations remained consistent in a subgroup analysis of separate surgical and endovascular cohorts and are depicted in detail in Table 4.

**Table 3:** Prediction of CVS and DCI in binary multivariate logistic regression.

	Cerebral Vasospams			Delayed Cerebral Ischemia		
*n* = 233	OR	95% CI	p-value	OR	95% CI	p-value
Therapy			0.072			0.086
endovascular	0.47	0.2, 1.07		0.61	0.34, 1.07	
surgical	—	—		—	—	
mFS			< 0.001**			0.15
1	—	—		—	—	
2	6.4	0.93, 64.1		2.86	0.63, 14.3	
3	5.65	1.70, 19.5		1.77	0.61, 5.91	
4	27.1	6.59, 126		2.98	1, 10.2	
TC decrease [%]			< 0.001**			0.003*
≤ 12.6	—	—		—	—	
> 12.6	10.6	4.74, 25.3		2.7	1.39, 5.43	

**Table 4:** Differences in prediction of CVS and DCI in surgical and endovascular group in a multivariate logistic regression.

	CVS prediction in surgical cohort			CVS prediction in endovascular cohort			DCI prediction in surgical cohort			DCI prediction in endovascular cohort		
	OR	95% CI	p-value	OR	95% CI	p-value	OR	95% CI	p-value	OR	95% CI	p-value
mFS												
1	—	—	—	—	—	—	—	—	—	—	—	—
2	—^+^	—^+^	—^+^	2.1	-2.45, 3.93	0.648	3.06	-0.79, 3.02	0.25	2.61	-1.95, 3.87	0.518
3	5.14	-0.18, 3.46	0.078	6.23	0.18, 3.48	0.030*	1.88	-0.87, 2.14	0.41	1.73	-1.11, 2.21	0.516
4	10.36	0.12, 4.56	0.039*	34.4	1.67, 5.41	< 0.001**	3.06	-0.47, 2.7	0.167	2.96	-0.58, 2.75	0.201
TC decrease [%]												
≤ 12.6	—	—	—	—	—	—	—	—	—	—	—	—
> 12.6	15.81	1.45, 4.07	< 0.001**	10.9	1.33, 3.45	< 0.001**	3.36	0.17, 2.25	0.023*	2.5	0.06, 1.77	0.036*

* *p* < 0.05; ** *p* < 0.001; ^+^unrealistically high values measured, since all grade 2 mFS patients in surgical cohort developed CVS.

*CVS* cerebral vasospasm, *DCI* delayed cerebral ischemia, *mFS* modified fisher scale, *TC* thrombocyte count, *Hct* hematocrit, *CRP* C-reactive-protein.

The predictive value of a relative thrombocyte count decrease > 12.6% for CVS and DCI was assessed using ROC analysis. The AUC was 0.76 for CVS (sensitivity 81.9%, specificity 70.6%) and 0.61 for DCI (sensitivity 83.3%, specificity 38.7%). These findings indicate good discriminatory power for CVS, while the moderate performance for DCI suggests additional contributing factors or different cut off value, which is discussed below (Fig. 2).

### Thrombocyte count decrease associated with DCI in a subgroup analysis of patients with CVS

**Fig. 5:**
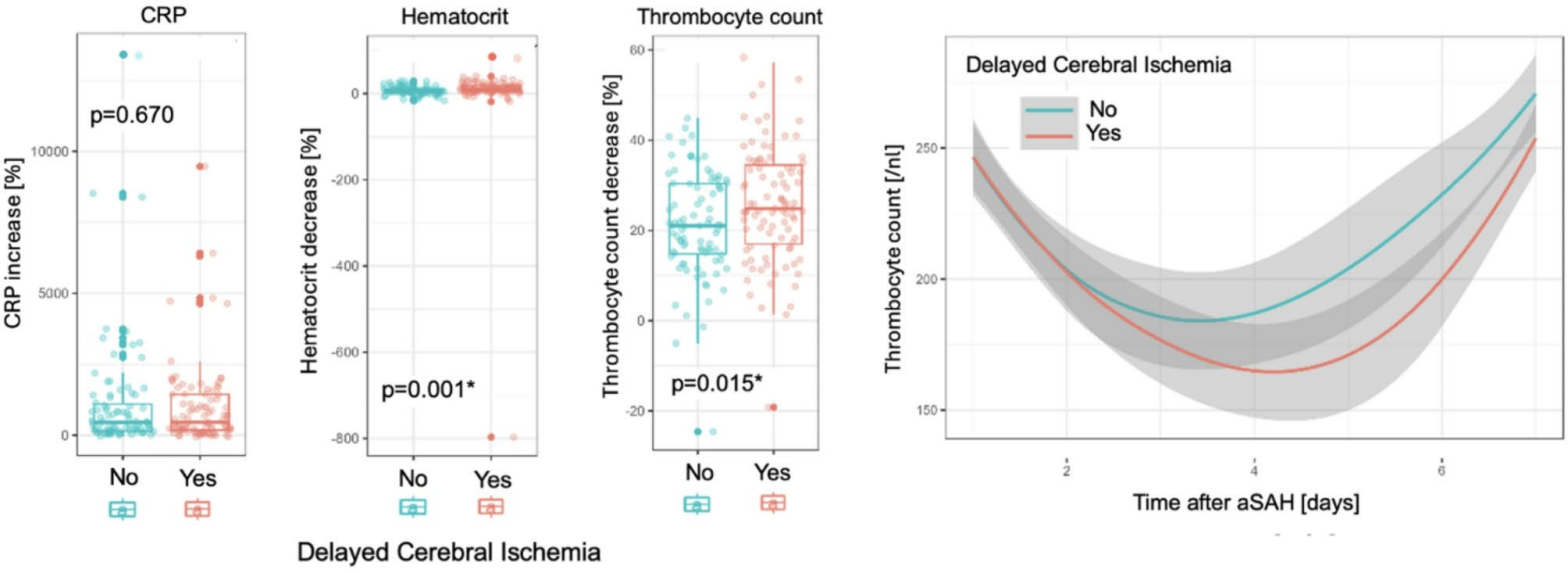
Subgroup analysis of patients with CVS on DCI presence Left: Box plots depicting results of Wilcoxon matched pair test showing differences in percentual changes of laboratory parameters grouped by DCI presence in CVS patients (no DCI = blue, DCI = red). Median, IQR with 25th and 75^Th^ quartile and maximums without outliers are presented. Only Hct and TC values showed significant differences, with Hct showing the most significant distinction as a sign of heterogeneity in hydration status between CVS patients with and without DCI. Right: Temporal follow-up [days after aSAH] of median TC values [/nl] with 95% CI in CVS patients grouped by DCI presence (no DCI = blue line, DCI = red line; line represents median) showing similar rate of TC decrease at the disease onset but overall lower TC reached in patients with DCI confirming the observations from the initial analysis. *CRP* C-reactive protein, *Hct* hematocrit, *TC* thrombocyte count, *DCI* delayed cerebral ischemia, *IQR* interquartile range, *CI* confidence interval.

Of 182 aSAH patients with CVS, 95 (52%) developed DCI. TC decrease was the only single predictor of DCI development both univariate (OR 1.03, CI [1.01–1.06], *p* < 0.013) and multivariate (OR 1.03, CI [1.01–1.06], *p* < 0.025). Age, sex, type of therapy, mFS, Hct decrease, and CRP increase showed no significant association with the development of DCI (Table 5). Only relative changes in TC and Hct showed significant differences between the two groups (*p* = 0.015 and *p* = 0.001 respectively; Fig.5 left). The TC dynamics in patients with CVS based on the presence of DCI show similar median TC values on day 1 after aSAH with a clear diversion of the median TC values between the two groups around day 2 and day 3 after aSAH (Fig. 5).

**Table 5:** DCI prediction in CVS patients in univariate and multivariate logistic regression.

*n* = 182	Univariate logistic regression			Multivariate logistic regression		
	OR	95% CI	p-value	OR	95% CI	p-value
Age	1	0.97, 1.02	0.77	0.99	0.97, 1.02	0.59
Sex			0.56			0.59
female	—	—		—	—	
male	0.83	0.45, 1.54		0.83	0.41, 1.65	
Therapy			0.24	0.99	0.97, 1.02	0.59
endovascular	0.71	0.39–1.26		0.40	0.16, 0.95	
surgical	—	—		—	—	
mFS			0.77			0.95
1	—	—		—	—	
2	1.40	0.23, 8.89		0.88	0.13, 6.24	
3	0.77	0.18, 3.07		0.69	0.15, 2.96	
4	0.95	0.22, 3.86		0.76	0.16, 3.43	
TC decrease [%]	1.03	1.01, 1.06	0.013*	1.03	1.00, 1.06	0.025*
Hct decrease [%]	1	0.99, 1	0.77	1	0.99, 1.01	0.98
CRP increase [%]	1	1, 1	0.83	1	1, 1	0.88

* *p* < 0.05; ** *p* < 0.001; ^+^unrealistically high values measured, since all grade 2 mFS patients in surgical cohort developed CVS.

*CVS* cerebral vasospasm, *DCI* delayed cerebral ischemia, *mFS* modified fisher scale, *TC* thrombocyte count, *Hct* hematocrit, *CRP* C-reactive-protein.

### Potential confounders and their influence on primary outcomes

Overall, 19 patients (8.5%) were taking ASA as part of their home medication. During interventions, 110 patients (47.2%) received intravenous ASA infusion, 11 (4.7%) received intravenous ticagrelor infusion, and 120 (51.5%) received intravenous UFH infusion. Significant differences were observed between patients with and without CVS and/or DCI regarding intrainterventional ASA administration (*p* = 0.028 for CVS and *p* = 0.027 for DCI) and UFH administration (*p* = 0.033 for CVS and *p* = 0.025 for DCI). The majority of these patients did not develop CVS and/or DCI. Meanwhile, the use of ticagrelor or NOACs showed no significant differences between the two subcohorts.

Regarding autoimmune and atopic diseases, only Hashimoto thyroiditis, bronchial asthma, and inflammatory bowel disease (IBD) were identified within our cohort. No significant differences in these conditions were observed between patients with and without CVS or DCI. Details on the distribution of these diseases, along with a detailed analysis of antiplatelet agents and anticoagulants, are summarized in Supplementary Table 1.

In univariate logistic regression, the intra-interventional intravenous administration of ASA and UFH was significantly associated with a lower relative risk of CVS (ASA: OR 0.49, CI [-1.34, -0.07], *p* = 0.03; UFH: OR 0.5, CI [-1.34, -0.05], *p* = 0.035). Similarly, these interventions were associated with a lower relative risk of DCI (ASA: OR 0.55, CI [-1.13, -0.07], *p* = 0.027; UFH: OR 0.55, CI [-1.13, -0.08], *p* = 0.025). However, these associations did not persist in multivariate logistic regression analyses. Additionally, no significant associations were found between autoimmune or atopic diseases and the risk of CVS or DCI (Supplementary Table 2).

To further investigate the influence of antiplatelet medications and anticoagulation on our findings, we conducted additional logistic regression analyses using our TC decrease cut-off as the dependent variable. In the univariate setting, ASA use as home medication (OR 0.45, CI [-1.4, -0.22], *p* = 0.007), its intrainterventional intravenous administration (OR 0.42, CI [-1.45, -0.29], *p* = 0.003), and the intrainterventional intravenous administration of UFH (OR 0.45, CI [-1.38, -0.22], *p* = 0.007) were all significantly associated with a TC decrease below 12.6%. However, these associations were not confirmed in the multivariate analysis (Supplementary Table 3).

## Discussion

In our study, an early decrease of TC shortly after aSAH was significantly predictive of CVS and DCI. The incidence of radiographically proven CVS in our cohort, in which induced hypertension therapy was initiated, was 78%, with 41% of patients developing DCI. Current literature reports the incidence of CVS and DCI as high as 70% and 30%, respectively^2,3^. Our study confirmed a higher risk of CVS in patients with higher mFS grade on admission. This was not observed for DCI, for which conflicting studies have been published^26,27^. The only independent variable in our study other than mFS significantly associated with CVS and DCI was the relative decrease in TC observed shortly after aSAH. Patients with a TC decrease greater than 12.6% had a 10-fold increased relative risk of CVS and a 3-fold increased risk of DCI. Interestingly, patients with a more profound decrease in TC tended to develop CVS earlier. In our cohort, endovascular treatment showed a minimal protective effect. This was not significant after dichotomization of our data based on TC decrease cut-off. Although mechanical manipulation of cerebral arteries during microsurgical clipping could theoretically lead to CVS, several previous studies with larger cohorts have reported no difference in association with CVS between endovascular and surgical treatment^28–30^. It could also be argued that surgery alone could lead to thrombocyte depletion and thus a decrease in TC. However, in a similar study, Hirashima et al. included a control group of elective cranioplasty cases without observing such a decrease in TC, making this observed association unlikely^31^.

As DCI and CVS remain the predominant causes of significant morbidity and mortality in patients after aSAH^1^, the need for risk stratification and implementation of reliable diagnostic tools to screen and detect such complications remains a top priority in the care of aSAH patients. Risk stratification based on mFS has been widely used as a predictor of CVS^23^. The most commonly used diagnostic tool to screen for CVS is TCD, which is dependent on operator experience, resulting in high interrater variability^32^. To our knowledge, there is no widely used predictive marker for CVS or DCI that reflects the pathophysiology of these complications.

The pathophysiology of CVS and DCI has been the subject of extensive research over the past three decades. Involvement of the inflammatory cascade has been proposed as a key mediator in the pathophysiology of CVS^33^. The observed association of increased expression of several cytokines^34^, e.g. the peak expression of CD34 at the time of CVS diagnosis^35^ in canine and mouse aSAH models, only further validates the inflammatory theory of the development of CVS and DCI. Thrombocytes also appear to play a critical role in the inflammatory cascade. A decrease in the plasma TC is a component of the acute phase response, hence the exclusion of patients diagnosed with sepsis and bacteremia from our study, as these clinical conditions massively influence plasma TC^36^. A disproportionate release of a thrombocyte prostaglandin, thromboxane A2 (TXA2), leads to extensive thrombocyte aggregation and vasoconstriction in the cerebral vasculature, which is exacerbated by the subsequent release of other vasoactive substances: adenosine diphosphate and serotonin^37^. The interventional study by Tokiyoshi et al. even showed that inhibition of TXA2 synthetase, which is responsible for the production of TXA2 in thrombocytes, reduced symptomatic vasospasm^38^. In turn, a dysregulated thrombocyte aggregation after aSAH may lead to microthrombosis, resulting in microcirculatory hypoperfusion and generation of DCI^39^. Indeed, for the formation of microthrombi, the abundant activation of coagulation cascade is needed. Specifically for primary hemostasis, the increasing release of platelet activation factor during the first 4 days after aSAH has already been reported^40^. The platelet activation is believed to contribute to the generation of DCI and was associated with more severe early brain injury and worse neurological outcome on 3-month follow-up^41^. The processes of inflammation and microthrombosis formation are probably intertwined and have potentiating effects on each other in their critical roles in DCI development^42^. Microthrombosis has also been postulated to contribute to the generation of cortical spreading depolarizations^43^. Our study therefore puts the role of thrombocyte depletion early after the aSAH in context of both, inflammatory and microthrombosis theories of CVS and DCI.

We could identify three studies investigating the observed decrease in TC in relation to CVS and/or DCI. Hirashima et al. retrospectively evaluated the decrease in TC as an independent risk factor for symptomatic CVS after aSAH. All were surgically treated patients. They found that the identified decrease in TC of more than 30% was significantly associated with the development of delayed ischemic neurological deficit (DIND) due to CVS^31^. In a prospective setting, Aggarval et al. studied 74 consecutive aSAH patients. Similarly, they found that the relative TC decrease detected between day 5 and day 9 after aSAH was significantly associated with DIND^44^. In a recent study from Rieß et al., thrombocytopenia was associated with higher DCI rates, higher in-hospital mortality and higher rate of non-favorable outcomes. Group of patients demonstrating a decrease in TC during the course of the disease also showed higher in-hospital mortality^45^. In our study, we also performed a time-to-event analysis, which showed a significantly higher rate of CVS in the group of patients with the observed decrease in TC. We also correlated the magnitude of this relative TC decrease with the time of CVS onset and found that patients with a greater relative TC decrease tended to develop CVS earlier. This association suggests a potential diagnostic window for early detection and intervention and should be subjected to validation in prospective studies.

Regarding the limitations of our study, the retrospective design is certainly the most limiting. Several potential confounders may not have been fully accounted for in our analysis. One notable factor is the standard treatment protocol for CVS, which includes induced hypertension. This intervention can contribute to hemodilution, potentially affecting thrombocyte counts. Hemodilution, however, is an inherent limitation in retrospective studies of this type. Additionally, aggressive fluid resuscitation, commonly used in the acute management of aSAH, may further influence thrombocyte counts through dilution effects. We also acknowledge the controversy of induced hypertension therapy, whose efficacy in treating CVS and DCI has not yet been confirmed in a randomized setting. The only RCT of induced hypertension in the treatment of DCI was prematurely terminated due to slow recruitment, but conveyed an important message that this therapy may be of benefit only in selected patients with aSAH, calling for better risk stratification of patients with CVS^46^. Despite this, our data indicate that hematocrit (Hct) changes were not significantly associated with CVS or DCI in multivariate regression analyses. While hydration status was assessed using Hct values, additional metrics, such as fluid balance data, would be necessary to fully account for this confounding variable. Another potential confounder, systemic inflammation, can lead to thrombocyte sequestration and depletion. The elevated C-reactive protein (CRP) levels observed in patients with CVS likely reflect an inflammatory response. To mitigate this confounding factor, we excluded patients with documented sepsis and bacteremia. Moreover, CRP itself showed no significant predictive value for DCI in our analysis, supporting the hypothesis that thrombocyte count (TC) dynamics are not solely driven by inflammation. Nonetheless, subclinical infections or transient inflammatory states—undetectable by CRP alone—could still influence thrombocyte counts. Future studies could use more sensitive inflammatory markers (e.g., interleukin-6 [IL-6], procalcitonin) or established clinical scores, such as the Sequential Organ Failure Assessment (SOFA) score, to better characterize this factor^47^. Pre-existing conditions, such as liver dysfunction, autoimmune disorders, or hematological diseases, could also contribute to baseline thrombocytopenia. We attempted to minimize this confounding effect by excluding patients with known hereditary thrombocyte conditions and analyzing the potential impact of autoimmune or atopic diseases, which were found to be insignificant. However, the influence of undiagnosed conditions cannot be entirely ruled out.

The role of TC dynamics in the development of CVS and DCI raises questions about the use of antiplatelet and anticoagulation therapy in aSAH patients. While not confirmed in multivariate analyses, our data suggested that intrainterventional administration of ASA and UFH in endovascularly treated patients was associated with a lower relative risk of CVS and DCI. However, because nearly all endovascularly treated patients at our center received both ASA and UFH intravenously, we could not isolate the specific effects of these agents from those of the coiling procedure itself. Furthermore, we observed no effect from orally administered ASA or DOACs taken as part of home medication. The existing literature offers some support for a protective role of ASA. For instance, long-term oral ASA use has been associated with lower mortality, improved functional outcomes, and potentially reduced DCI incidence^48^. Garton et al. similarly reported an association between ASA use and lower DCI rates, as well as better functional outcomes^49^. However, both findings stem from meta-analyses dominated by retrospective studies with limited reproducibility, warranting cautious interpretation. A randomized controlled trial (RCT) examining the use of 100 mg ASA as a suppository for 14 days following aSAH was discontinued after interim analyses suggested negligible effects on DCI development^50^. Regarding UFH, its impact on CVS and DCI risk remains poorly understood due to the lack of randomized studies. An ongoing RCT is currently evaluating the effects of low-dose intravenous UFH on these outcomes^51^. In our cohort, all aSAH patients received low-molecular-weight heparin (LMWH), primarily enoxaparin, starting 24 h after ictus, precluding an assessment of its effects from our data. However, an RCT by Siironen et al. found no significant effect of LMWH on the functional outcomes of aSAH patients^52^. There are currently over 300 drugs that may potentially cause drug-induced thrombocytopenia (DITP). Of those most frequently administered ones in patients after aSAH are already mentioned LMWH, antiplatelet drugs, antibiotics (especially piperacillin, ceftriaxone and vancomycin) and non-steroid anti-inflammatory drugs (NSAIDs, e.g. ibuprofen). DITP caused by antibiotics and NSAIDs is an extremely rare condition with only case reports available^53^. Moreover, the vast majority of our patients with or without CVS and/or DCI never reached levels of TC below a threshold of a thrombocytopenia. Since no effect of broadly administered antiplatelet agents and UFH on TC decrease was found, no further analysis on the remaining drugs rarely associated with DITP was performed. Despite this, we cannot fully rule out the influence of these or other broadly administered medications on our data.

We are also aware that the actual cut-off value for the TC decrease in DCI might differ from that in CVS, especially when studies showing discrepancy between processes involved in CVS and DCI, suggesting DCI development independent of CVS, are taken into account^54^. However, the reason for choosing the same cut-off value for both, CVS and DCI, was the inability to reliably determine the onset of DCI, especially in a retrospective setting. Our study also lacks a clinical correlate of the developed CVS and DCI (DIND) or outcome measures at follow-up. We believe that these are best studied in a prospective, standardized fashion to ensure sufficient reliability of such data inasmuch going beyond the scope of our study.

The causal relationship between TC decrease and CVS/DCI development remains uncertain, underscoring the need for further prospective mechanistic studies. If such prospective studies confirm our findings, it might potentially provide a relatively simple marker for the risk stratification of patients after aSAH.

## Conclusions

In conclusion, our study highlights the potential importance of thrombocyte count dynamics in the context of CVS and DCI following aSAH. Specifically, we observed that a relative decrease in TC greater than 12.6% within the first few days following aSAH was strongly associated with the development of CVS and DCI, often preceding their radiographic diagnosis by a median of three days. However, it is important to emphasize that these findings are associative and require confirmation in prospective studies. If validated, TC dynamics could represent an early indicator of CVS and DCI risk stratification, potentially informing the timing of interventions to reduce associated morbidity and mortality.

## Electronic supplementary material

Below is the link to the electronic supplementary material.

Supplementary Material 1

Supplementary Material 2

Supplementary Material 3

## Data Availability

The data supporting the findings of this study are not publicly available due to local GDPR regulations. However, access to this data can be granted upon reasonable request via email (Oros@med.uni-frankfurt.de).

## References

[1] Broderick, J. P., Brott, T. G., Duldner, J. E., Tomsick, T. & Leach, A. Initial and recurrent bleeding are the major causes of death following subarachnoid hemorrhage. 10.1161/01.str.25.7.1342 8023347

[2] Dorsch, N. W. C. & King, M. T. A review of cerebral vasospasm in aneurysmal subarachnoid haemorrhage part I: incidence and effects. *J. Clin. Neurosci.* **1** (1), 19–26. 10.1016/0967-5868(94)90005-1 (1994). 18638721 10.1016/0967-5868(94)90005-1

[3] Ferguson, S., Macdonald, R. L.,Predictors of cerebral & infarction in patients with aneurysmal subarachnoid hemorrhage. *Neurosurgery* ;**60**(4):658–667. doi:10.1227/01.NEU.0000255396.23280.31 (2007). 17415202 10.1227/01.NEU.0000255396.23280.31

[4] Frontera, J. A. et al. Defining vasospasm after subarachnoid hemorrhage: what is the most clinically relevant definition?? *Stroke* **40** (6), 1963–1968. 10.1161/STROKEAHA.108.544700 (2009). 19359629 10.1161/STROKEAHA.108.544700

[5] Matamoros, C. E. S. et al. Prediction of symptomatic vasospasm in patients with aneurysmal subarachnoid hemorrhage using early transcranial doppler. 19 . PMC6998809 32071668

[6] Toi, H. et al. Prediction of cerebral vasospasm using early stage transcranial doppler. *Neurol. Med. Chir. (Tokyo)*. **53** (6), 396–402. 10.2176/nmc.53.396 (2013). 23803618 10.2176/nmc.53.396

[7] Jaeger, M., Schuhmann, M. U., Soehle, M., Nagel, C. & Meixensberger, J. Continuous monitoring of cerebrovascular autoregulation after subarachnoid hemorrhage by brain tissue oxygen pressure reactivity and its relation to delayed cerebral infarction. *Stroke* **38** (3), 981–986. 10.1161/01.STR.0000257964.65743.99 (2007). 17272764 10.1161/01.STR.0000257964.65743.99

[8] Lang, E. W., Mulvey, J. M., Mudaliar, Y. & Dorsch, N. W. C. Direct cerebral oxygenation monitoring—a systematic review of recent publications. *Neurosurg. Rev.* **30** (2), 99–107. 10.1007/s10143-006-0062-4 (2007). 17221264 10.1007/s10143-006-0062-4

[9] Winberg, J. et al. Cerebral Microdialysis-Based interventions targeting delayed cerebral ischemia following aneurysmal subarachnoid hemorrhage. *Neurocrit Care*. **37** (1), 255–266. 10.1007/s12028-022-01492-5 (2022). 35488171 10.1007/s12028-022-01492-5 PMC9283139

[10] Gabikian, P. et al. Prevention of experimental cerebral vasospasm by intracranial delivery of a nitric oxide donor from a Controlled-Release polymer: toxicity and efficacy studies in rabbits and rats. *Stroke* **33** (11), 2681–2686. 10.1161/01.STR.0000033931.62992.B1 (2002). 12411661 10.1161/01.str.0000033931.62992.b1

[11] Santhanam, A. V. R. et al. Role of endothelial NO synthase phosphorylation in cerebrovascular protective effect of Recombinant erythropoietin during subarachnoid Hemorrhage– induced cerebral vasospasm. *Stroke* **36** (12), 2731–2737. 10.1161/01.STR.0000190021.85035.5b (2005). 16269632 10.1161/01.STR.0000190021.85035.5b

[12] Jung, C. S. et al. Association of an endogenous inhibitor of nitric oxide synthase with cerebral vasospasm in patients with aneurysmal subarachnoid hemorrhage. *J. Neurosurg.* **107** (5), 945–950. 10.3171/JNS-07/11/0945 (2007). 17977265 10.3171/JNS-07/11/0945

[13] Iuliano, B. A., Pluta, R. M., Jung, C. & Oldfield, E. H. Endothelial dysfunction in a primate model of cerebral vasospasm. *J. Neurosurg.* **100** (2), 287–294. 10.3171/jns.2004.100.2.0287 (2004). 15086237 10.3171/jns.2004.100.2.0287

[14] Juvela, S. Plasma endothelin concentrations after aneurysmal subarachnoid hemorrhage. *J. Neurosurg.* **92** (3), 390–400. 10.3171/jns.2000.92.3.0390 (2000). 10701524 10.3171/jns.2000.92.3.0390

[15] Mascia, L. et al. Temporal relationship between Endothelin-1 concentrations and cerebral vasospasm in patients with aneurysmal subarachnoid hemorrhage. 10.1161/01.str.32.5.1185 11340231

[16] Ohkuma, H., Itoh, K., Shibata, S. & Suzuki, S. Morphological changes of intraparenchymal arterioles after experimental subarachnoid hemorrhage in dogs. *Neurosurgery* **41** (1), 230–236. 10.1097/00006123-199707000-00036 (1997). 9218311 10.1097/00006123-199707000-00036

[17] Frijns, C. J. M. Early Circulating levels of endothelial cell activation markers in aneurysmal subarachnoid haemorrhage: associations with cerebral ischaemic events and outcome. *J. Neurol. Neurosurg. Psychiatry*. **77** (1), 77–83. 10.1136/jnnp.2005.064956 (2006). 16361599 10.1136/jnnp.2005.064956 PMC2117384

[18] Dreier, J. P. Vasospasm-Induced spreading depolarization and/or spreading-depolarization-induced vasospasm after subarachnoid hemorrhage. *Neurocrit Care*. **37** (S1), 5–7. 10.1007/s12028-021-01373-3 (2022). 34704217 10.1007/s12028-021-01373-3 PMC9259518

[19] Hirashima, Y. et al. Platelet-activating factor and cerebral vasospasm following subarachnoid hemorrhage. *J. Neurosurg.* **78** (4), 592–597. 10.3171/jns.1993.78.4.0592 (1993). 8450333 10.3171/jns.1993.78.4.0592

[20] Hiroshima, Y. et al. Elevation of platelet activating factor, inflammatory cytokines, and coagulation factors in the internal jugular vein of patients with subarachnoid hemorrhage. 10.1023/a:1021985030331 9342729

[21] Friedrich, V., Flores, R., Muller, A. & Sehba, F. A. Luminal platelet aggregates in functional deficits in parenchymal vessels after subarachnoid hemorrhage. *Brain Res.* **1354**, 179–187. 10.1016/j.brainres.2010.07.040 (2010). 20654597 10.1016/j.brainres.2010.07.040 PMC2933941

[22] Sehba, F. A., Mostafa, G., Friedrich, V. & Bederson, J. B. Acute microvascular platelet aggregation after subarachnoid hemorrhage. *J. Neurosurg.* **102** (6), 1094–1100. 10.3171/jns.2005.102.6.1094 (2005). 16028769 10.3171/jns.2005.102.6.1094

[23] Frontera, J. A. et al. Prediction of symptomatic vasospasmafter subarachnoid hemorrhage: the modified fisher scale. *Neurosurgery* **59** (1), 21–27. 10.1227/01.NEU.0000218821.34014.1B (2006). 16823296 10.1227/01.neu.0000243277.86222.6c

[24] Phan, K. et al. Ultra-Early angiographic vasospasm after aneurysmal subarachnoid hemorrhage: A systematic review and Meta-Analysis. *World Neurosurg.* **102**, 632–638e1. 10.1016/j.wneu.2017.03.057 (2017). 28365434 10.1016/j.wneu.2017.03.057

[25] Al-Mufti, F. et al. Ultra-early angiographic vasospasm associated with delayed cerebral ischemia and infarction following aneurysmal subarachnoid hemorrhage. *J. Neurosurg.* **126** (5), 1545–1551. 10.3171/2016.2.JNS151939 (2017). 27231975 10.3171/2016.2.JNS151939

[26] Eagles, M. E., Jaja, B. N. R. & Macdonald, R. L. Incorporating a modified Graeb score to the modified fisher scale for improved risk prediction of delayed cerebral ischemia following aneurysmal subarachnoid hemorrhage. *Neurosurgery* **82** (3), 299–305. 10.1093/neuros/nyx165 (2018). 28419304 10.1093/neuros/nyx165

[27] Van Der Steen, W. E. et al. Radiological scales predicting delayed cerebral ischemia in subarachnoid hemorrhage: systematic review and meta-analysis. *Neuroradiology* **61** (3), 247–256. 10.1007/s00234-019-02161-9 (2019). 30693409 10.1007/s00234-019-02161-9

[28] Ibrahim, G. M., Morgan, B. R. & Macdonald, R. L. Patient phenotypes associated with outcomes after aneurysmal subarachnoid hemorrhage: A principal component analysis. *Stroke* **45** (3), 670–676. 10.1161/STROKEAHA.113.003078 (2014). 24425125 10.1161/STROKEAHA.113.003078

[29] Gross, B. A., Rosalind Lai, P. M., Frerichs, K. U. & Du, R. Treatment modality and vasospasm after aneurysmal subarachnoid hemorrhage. *World Neurosurg.* **82** (6), e725–e730. 10.1016/j.wneu.2013.08.017 (2014). 23954735 10.1016/j.wneu.2013.08.017

[30] Rabinstein, A. A. et al. Symptomatic vasospasm and outcomes following aneurysmal subarachnoid hemorrhage: a comparison between surgical repair and endovascular coil occlusion. *J. Neurosurg.* **98** (2), 319–325. 10.3171/jns.2003.98.2.0319 (2003). 12593618 10.3171/jns.2003.98.2.0319

[31] Hirashima, Y., Hamada, H., Kurimoto, M., Origasa, H. & Endo, S. Decrease in platelet count as an independent risk factor for symptomatic vasospasm following aneurysmal subarachnoid hemorrhage. *J. Neurosurg.* **102** (5), 882–887. 10.3171/jns.2005.102.5.0882 (2005). 15926714 10.3171/jns.2005.102.5.0882

[32] Melinosky, C. et al. The modified fisher scale lacks interrater reliability. *Neurocrit Care*. **35** (1), 72–78. 10.1007/s12028-020-01142-8 (2021). 33200331 10.1007/s12028-020-01142-8

[33] Carr, K. R., Zuckerman, S. L., Mocco, J. & Inflammation Cerebral vasospasm, and evolving theories of delayed cerebral ischemia. *Neurol. Res. Int.* **2013**, 1–12. 10.1155/2013/506584 (2013). 10.1155/2013/506584 PMC3766617 24058736

[34] Aihara, Y., Kasuya, H., Onda, H., Hori, T. & Takeda, J. Quantitative analysis of gene expressions related to inflammation in canine spastic artery after subarachnoid hemorrhage. *Stroke* **32** (1), 212–217. 10.1161/01.STR.32.1.212 (2001). 11136939 10.1161/01.str.32.1.212

[35] Wang, Z. et al. Potential role of CD34 in cerebral vasospasm after experimental subarachnoid hemorrhage in rats. *Cytokine* **52** (3), 245–251. 10.1016/j.cyto.2010.08.002 (2010). 20829062 10.1016/j.cyto.2010.08.002

[36] Vardon-Bounes, F. et al. Platelets are critical key players in sepsis. *Int. J. Mol. Sci.* **20** (14), 3494. 10.3390/ijms20143494 (2019). 31315248 10.3390/ijms20143494 PMC6679237

[37] Ellis, E. F., Nies, A. S. & Oates, J. A. Cerebral arterial smooth muscle contraction by thromboxane A2. *Stroke* **8** (4), 480–483. 10.1161/01.STR.8.4.480 (1977). 898244 10.1161/01.str.8.4.480

[38] Tokiyoshi, K., Ohnishi, T. & Nii, Y. Efficacy and toxicity of thromboxane synthetase inhibitor for cerebral vasospasm after subarachnoid hemorrhage. *Surg. Neurol.* **36** (2), 112–118. 10.1016/0090-3019(91)90228-2 (1991). 1891755 10.1016/0090-3019(91)90228-2

[39] Vergouwen, M. D., Vermeulen, M., Coert, B. A., Stroes, E. S. & Roos, Y. B. Microthrombosis after aneurysmal subarachnoid hemorrhage: an additional explanation for delayed cerebral ischemia. *J. Cereb. Blood Flow. Metab.* **28** (11), 1761–1770. 10.1038/jcbfm.2008.74 (2008). 18628782 10.1038/jcbfm.2008.74

[40] Rowland, M. J., Hadjipavlou, G., Kelly, M., Westbrook, J. & Pattinson, K. T. S. Delayed cerebral ischaemia after subarachnoid haemorrhage: looking beyond vasospasm. *Br. J. Anaesth.* **109** (3), 315–329. 10.1093/bja/aes264 (2012). 22879655 10.1093/bja/aes264

[41] Frontera, J. A. et al. The role of platelet activation and inflammation in early brain injury following subarachnoid hemorrhage. *Neurocrit Care*. **26** (1), 48–57. 10.1007/s12028-016-0292-4 (2017). 27430874 10.1007/s12028-016-0292-4 PMC6354928

[42] McBride, D. W., Blackburn, S. L., Peeyush, K. T., Matsumura, K. & Zhang, J. H. The role of thromboinflammation in delayed cerebral ischemia after subarachnoid hemorrhage. *Front. Neurol.* **8**, 555. 10.3389/fneur.2017.00555 (2017). 29109695 10.3389/fneur.2017.00555 PMC5660311

[43] Dietrich, W. D., Feng, Z. C., Leistra, H., Watson, B. D. & Rosenthal, M. Photothrombotic infarction triggers multiple episodes of cortical spreading depression in distant brain regions. *J. Cereb. Blood Flow. Metab.* **14** (1), 20–28. 10.1038/jcbfm.1994.4 (1994). 8263054 10.1038/jcbfm.1994.4

[44] Aggarwal, A. et al. Vasospasm following aneurysmal subarachnoid hemorrhage: thrombocytopenia a marker. *J. Neurosci. Rural Pract.* **04** (03), 257–261. 10.4103/0976-3147.118762 (2013). 10.4103/0976-3147.118762 PMC3821408 24250155

[45] Rieß, C. et al. Baseline and average platelet count can predict the outcome of patients with aneurysmal subarachnoid hemorrhage. *World Neurosurg. X*. **22**, 100302. 10.1016/j.wnsx.2024.100302 (2024). 39790119 10.1016/j.wnsx.2024.100302 PMC11711821

[46] Gathier, C. S. et al. Induced hypertension for delayed cerebral ischemia after aneurysmal subarachnoid hemorrhage: A randomized clinical trial. *Stroke* **49** (1), 76–83. 10.1161/STROKEAHA.117.017956 (2018). 29158449 10.1161/STROKEAHA.117.017956

[47] Singer, M. et al. The third international consensus definitions for sepsis and septic shock (Sepsis-3). *JAMA* **315** (8), 801. 10.1001/jama.2016.0287 (2016). 26903338 10.1001/jama.2016.0287 PMC4968574

[48] Zhao, L. et al. Effect of antiplatelet treatment on aneurysmal subarachnoid hemorrhage patients after endovascular treatment: a systematic review with meta-analysis. *Neurosurg. Rev.* **45** (6), 3523–3536. 10.1007/s10143-022-01877-2 (2022). 36178562 10.1007/s10143-022-01877-2

[49] Garton, A. L. A. et al. Antiplatelet therapy and outcomes after aneurysmal subarachnoid hemorrhage: A systematic review and meta-analysis. *Clin. Neurol. Neurosurg.* **235**, 108025. 10.1016/j.clineuro.2023.108025 (2023). 37925994 10.1016/j.clineuro.2023.108025 PMC10841860

[50] Van Den Bergh, W. M. Randomized controlled trial of acetylsalicylic acid in aneurysmal subarachnoid hemorrhage: the MASH study. *Stroke* **37** (9), 2326–2330. 10.1161/01.STR.0000236841.16055.0f (2006). 16888270 10.1161/01.STR.0000236841.16055.0f

[51] Zhang, Y. & Hu, J. Effects of low-dose intravenous heparin therapy in aneurysmal subarachnoid hemorrhage: a randomized controlled clinical trial protocol. *Trials* **24** (1), 447. 10.1186/s13063-023-07493-9 (2023). 37422666 10.1186/s13063-023-07493-9 PMC10329784

[52] Siironen, J. et al. No effect of Enoxaparin on outcome of aneurysmal subarachnoid hemorrhage: a randomized, double-blind, placebo-controlled clinical trial. *J. Neurosurg.* **99** (6), 953–959. 10.3171/jns.2003.99.6.0953 (2003). 14705720 10.3171/jns.2003.99.6.0953

[53] Vayne, C. et al. Pathophysiology and diagnosis of Drug-Induced immune thrombocytopenia. *J. Clin. Med.* **9** (7), 2212. 10.3390/jcm9072212 (2020). 32668640 10.3390/jcm9072212 PMC7408966

[54] Roos, Y. B. W. E. M. Complications and outcome in patients with aneurysmal subarachnoid haemorrhage: a prospective hospital based cohort study in the Netherlands. *J. Neurol. Neurosurg. Psychiatry*. **68** (3), 337–341. 10.1136/jnnp.68.3.337 (2000). 10675216 10.1136/jnnp.68.3.337 PMC1736841

