# Correction: Use of thrombocyte count dynamics after aneurysmal subarachnoid hemorrhage to predict cerebral vasospasm and delayed cerebral ischemia: a retrospective monocentric cohort study

**DOI:** 10.1038/s41598-025-07305-x

**Published:** 2025-07-15

**Authors:** Jan Oros, Stefanos Voglis, Ferdinand Oliver Bohmann, Lina Elisabeth Qasem, Christophe Théo Arendt, Fee Keil, Wolfgang Miesbach, Marcus Czabanka, Sarah Christina Reitz

**Affiliations:** 1 Center for Neurology and Neurosurgery, Department of Neurosurgery, Goethe University Frankfurt, University Hospital, Frankfurt Am Main, Germany; 2 Department of Neurosurgery, University of Zürich, University Hospital, Zürich, Switzerland; 3 Center for Neurology and Neurosurgery, Department of Neurology, Goethe University Frankfurt, University Hospital, Frankfurt Am Main, Germany; 4 Institute of Neuroradiology, Goethe University Frankfurt, University Hospital, Frankfurt Am Main, Germany; 5 Department of Hematology and Oncology, Goethe University Frankfurt, University Hospital, Frankfurt Am Main, Germany

Correction to: *Scientific Reports* 10.1038/s41598-025-93767-y, published online 21 March 2025

In the original version of this Article Ferdinand Oliver Bohmann was incorrectly affiliated with ‘Center for Neurology and Neurosurgery, Department of Neurosurgery, Goethe University Frankfurt, University Hospital, Frankfurt am Main, Germany’. The correct affiliation is listed below.

Center for Neurology and Neurosurgery, Department of Neurology, Goethe University Frankfurt, University Hospital, Frankfurt am Main, Germany.

The original Article has been corrected.

