# Supplementary material for: Use of thrombocyte count dynamics after aneurysmal subarachnoid hemorrhage to predict cerebral vasospasm and delayed cerebral ischemia: a retrospective monocentric cohort study
Source: Sci Rep. 2025 Mar 21;15:9826. doi: 10.1038/s41598-025-93767-y (PMC11928525; doi:10.1038/s41598-025-93767-y)
Supplement: Supplementary file 1 — Supplementary Material 1 [file 41598_2025_93767_MOESM1_ESM.docx]

| **Supplementary Table 3** Logistic regressions on influence of APT and anticoagulation on TC decrease | | | | | | |
| --- | --- | --- | --- | --- | --- | --- |
|  | **Thrombocyte count decrease > 12.6%** | | | | | |
|  | **Univariate logistic regression** | | | **Multivariate logistic regression** | | |
| **n = 233** | **OR** | **95% CI** | **p-value** | **OR** | **95% CI** | **p-value** |
| ***APT*** |  |  |  |  |  |  |
| ASA H/M | 0.45 | -1.40, -0.22 | **0.007*** | 0.43 | -2.25, 0.58 | 0.248 |
| ASA I/I | 0.42 | -1.45, -0.29 | **0.003*** | 0.21 | -3.43, 0.28 | 0.095 |
| Ticagrelor I/I | 0.49 | -1.94, 0.5 | 0.247 | 0.78 | -1.52, 1.02 | 0.701 |
| ***Anticoagulation*** |  |  |  |  |  |  |
| UFH I/I | 0.45 | -1.38, -0.22 | **0.007*** | 1.92 | -1.14, 2.45 | 0.475 |
| NOAC | 1.28 | -1.39, 1.87 | 0.772 | 1.77 | -1.07, 2.24 | 0.488 |
| ***** p < 0.05  TC = thrombocyte count, APT = antiplatelet therapy, ASA = acetylsalicylic acid, H/M home medication, I/I = intrainterventional, UFH = unfractioned heparin, NOAC = novel oral anticoagulation | | | | | | |
