# Supplementary material for: Use of thrombocyte count dynamics after aneurysmal subarachnoid hemorrhage to predict cerebral vasospasm and delayed cerebral ischemia: a retrospective monocentric cohort study
Source: Sci Rep. 2025 Mar 21;15:9826. doi: 10.1038/s41598-025-93767-y (PMC11928525; doi:10.1038/s41598-025-93767-y)
Supplement: Supplementary file 2 — Supplementary Material 2 [file 41598_2025_93767_MOESM2_ESM.docx]

| **Supplementary Table 1** Basic characteristics of possible confounders with ability to influence the thrombocyte count | | | | | | | |
| --- | --- | --- | --- | --- | --- | --- | --- |
|  | **n= 233*^1^*** | **Cerebral Vasospasm** | | | **Delayed Cerebral Ischemia** | | |
|  |  | no, n = 51 (21.9%)*^1^* | yes, n = 182 (71.1%)*^1^* | **p-value*^2^*** | no, n = 137 (58.8%)^1^ | yes, n = 96 (41.2%)^1^ | **p-value*^2^*** |
| **APT** |  |  |  |  |  |  |  |
| **ASA H/M** |  |  |  | p=0.927 |  |  | p=0.569 |
| yes | 19 (8.5%) | 4 (7.8%) | 15 (8.2%) |  | 10 (7.3%) | 9 (9.4%) |  |
| no | 214 (91.5%) | 47 (92.2%) | 167 (91.8%) |  | 127 (92.7%) | 87 (90.6%) |  |
| **ASA I/I** |  |  |  | **p=0.028*** |  |  | **p=0.027*** |
| yes | 110 (47.2) | 31 (60.8%) | 79 (43.4%) |  | 73 (53.3%) | 37 (38.5%) |  |
| no | 123 (52.8) | 20 (39.2%) | 103 (56.6%) |  | 64 (46.7%) | 59 (61.5%) |  |
| **Ticagrelor I/I** |  |  |  | p=0.053 |  |  | p=0.738 |
| yes | 11 (4.7%) | 5 (9.8%) | 6 (3.3%) |  | 7 (5.1%) | 4 (4.2%) |  |
| no | 222 (95.3%) | 46 (90.2%) | 176 (96.7%) |  | 130 (94.9%) | 92 (95.8%) |  |
| **Anticoagulation** |  |  |  |  |  |  |  |
| **NOAC H/M** |  |  |  | p=0.829 |  |  | p=0.343 |
| yes | 8 (3.4%) | 2 (3.9%) | 6 (3.3%) |  | 6 (4.4%) | 2 (2.1%) |  |
| no | 225 (96.6%) | 49 (96.1%) | 176 (96.7%) |  | 131 (95.6%) | 94 (97.9%) |  |
| **UFH I/I** |  |  |  | **p=0.033*** |  |  | **p=0.025*** |
| yes | 120 (51.5%) | 33 (64.7%) | 87 (47.8%) |  | 79 (57.7%) | 41 (42.7%) |  |
| no | 113 (48.5%) | 18 (35.3%) | 95 (52.2%) |  | 58 (42.3%) | 55 (57.3%) |  |
| **Autoimmune/atopic diseases** |  |  |  |  |  |  |  |
| **HT** |  |  |  | p=0.572 |  |  | p=0.551 |
| yes | 38 (16.3%) | 7 (13.7%) | 31 (17%) |  | 24 (17.5%) | 14 (14.6%) |  |
| no | 195 (83.7%) | 44 (86.3%) | 151 (83%) |  | 113 (82.5%) | 82 (85.4%) |  |
| **BA** |  |  |  | p=0.322 |  |  | p=0.33 |
| yes | 5 (2.2%) | 2 (3.9%) | 3 (1.7%) |  | 4 (2.9%) | 1 (1.1%) |  |
| no | 228 (97.8%) | 49 (96.1%) | 179 (98.3%) |  | 133 (97.1%) | 95 (98.9%) |  |
| **IBD** |  |  |  | p=0.334 |  |  | p=0.8 |
| yes | 2 (0.9%) | 1 (1.9%) | 1 (0.6%) |  | 1 (0.7%) | 1 (1.1%) |  |
| no | 231 (99.1%) | 50 (98.1%) | 181 (99.4%) |  | 136 (99.3%) | 95 (98.9%) |  |
| ^1^Statistics presented: n (%)  ^2^Pearson's Chi-squared test (for all n ≥ 5) and Fisher's exact test (for all n < 5) for categorical variables  ***** p < 0.05  APT = antiplatelet therapy, ASA = acetylsalicylic acid, H/M = home medication, I/I = intrainterventional, UFH = unfractioned heparin, NOAC = novel oral anticoagulation, HT = Hashimoto thyroiditis, BA = bronchial asthma, IBD = inflammatory bowel disease | | | | | | | |
