# Supplementary material for: Use of thrombocyte count dynamics after aneurysmal subarachnoid hemorrhage to predict cerebral vasospasm and delayed cerebral ischemia: a retrospective monocentric cohort study
Source: Sci Rep. 2025 Mar 21;15:9826. doi: 10.1038/s41598-025-93767-y (PMC11928525; doi:10.1038/s41598-025-93767-y)
Supplement: Supplementary file 3 — Supplementary Material 3 [file 41598_2025_93767_MOESM3_ESM.docx]

| **Supplementary Table 2** Logistic regressions on influence of possible confounders on primary outcomes | | | | | | | | | | | | |
| --- | --- | --- | --- | --- | --- | --- | --- | --- | --- | --- | --- | --- |
|  | **Cerebral Vasospasm** | | | | | | **Delayed Cerebral Ischemia** | | | | | |
|  | **Univariate logistic regression** | | | **Multivariate logistic regression** | | | **Univariate logistic regression** | | | **Multivariate logistic**  **regression** | | |
| **n = 233** | **OR** | **95% CI** | **p-value** | **OR** | **95% CI** | **p-value** | **OR** | **95% CI** | **p-value** | **OR** | **95% CI** | **p-value** |
| ***APT*** |  |  |  |  |  |  |  |  |  |  |  |  |
| ASA H/M | 1.06 | -1.09, 1.20 | 0.927 | 0.92 | -1.77, 1.59 | 0.923 | 1.31 | -0.67, 1.21 | 0.57 | 1.58 | 0.97, 1.01 | 0.516 |
| ASA I/I | 0.49 | -1.34, -0.07 | **0.03*** | 0.65 | -2.54, 1.67 | 0.687 | 0.55 | -1.13, -0.07 | **0.027*** | 1.15 | -0.92, 1.83 | 0.875 |
| Ticagrelor I/I | 0.31 | -2.39, 0.07 | 0.065 | 0.43 | -2.12, 0.43 | 0.194 | 0.81 | -1.47, 1.04 | 0.739 | 1.13 | -1.19, 1.43 | 0.86 |
| ***Anticoagulation*** |  |  |  |  |  |  |  |  |  |  |  |  |
| UFH I/I | 0.50 | -1.34, -0.05 | **0.035*** | 0.81 | -2.27, 1.84 | 0.837 | 0.55 | -1.13, -0.08 | **0.025*** | 0.48 | -2.43, 0.94 | 0.387 |
| NOAC | 0.84 | -1.81, 1.45 | 0.829 | 1.14 | -1.54, 1.81 | 0.876 | 0.47 | -2.39, 0.86 | 0.354 | 0.59 | -2.17, 1.13 | 0.535 |
| ***Autoimmune/***  ***atopic disease*** |  |  |  |  |  |  |  |  |  |  |  |  |
| HT | 1.29 | -0.63, 1.14 | 0.573 | — | — | — | 0.80 | -0.94, 0.5 | 0.551 | — | — | — |
| BA | 0.41 | -2.71, 0.93 | 0.337 | — | — | — | 0.35 | -3.26, 1.16 | 0.351 | — | — | — |
| IBD | 0.28 | -4.08, 1.50 | 0.366 | — | — | — | 1.43 | -2.43, 3.14 | 0.801 | — | — | — |
| ***** p < 0.05  APT = antiplatelet therapy, ASA = acetylsalicylic acid, H/M home medication, I/I = intrainterventional, UFH = unfractioned heparin, NOAC = novel oral anticoagulation, HT = Hashimoto thyroiditis, BA = bronchial asthma, IBD = inflammatory bowel disease | | | | | | | | | | | | |
